# The Age at Which a Woman Becomes a Mother and Her Satisfaction with the Process of Pregnancy, Childbirth and the Puerperium

**DOI:** 10.3390/healthcare8020082

**Published:** 2020-04-01

**Authors:** Leticia Molina-García, Manuel Hidalgo-Ruiz, Alberto Gálvez-Toro, Silvia Cristina Aguilar-Puerta, Miguel Delgado-Rodríguez, Juan Miguel Martínez-Galiano

**Affiliations:** 1 Obstetrics and Gynecology Department, Jaen Hospital Complex, 23007 Jaen, Spain; letitedi@hotmail.com (L.M.-G.); atoro@telefonica.net (A.G.-T.); 2 Obstetrics and Gynecology Department, Hospital San Juan de la Cruz, 23400 Úbeda, Spain; manuel9282@gmail.com; 3 Obstetrics and Gynecology Department, Hospital Virgen de las Nieves, 18014 Granada, Spain; silviacap1984@gmail.com; 4 Department of Health Sciences, University of Jaen, 23071 Jaen, Spain; mdelgado@ujaen.es; 5 Consortium for Biomedical Research in Epidemiology and Public Health (CIBERESP), 28029 Madrid, Spain; 6 Department of Nursing, University of Jaen, 23071 Jaen, Spain

**Keywords:** maternal age, satisfaction, pregnancy, childbirth, health system, midwife care

## Abstract

This study assessed the effect of maternal age on satisfaction at each stage of pregnancy, childbirth and puerperium, and globally. An observational study was carried out in five hospitals of the Andalusian public health system with older primiparous women, from May 2016 to May 2018. Using a pre-piloted questionnaire, information was collected on pregnancy, childbirth, puerperium, newborn variables and degree of satisfaction with the care received. Crude and adjusted mean differences and the standard error of the mean were calculated. A total of 373 women participated. In total, 43.0% of the sample were very satisfied with the care received during pregnancy, and 74.2% with the care received during childbirth. During the puerperium, the highest percentage (60.4%) was found among the women who reported being quite satisfied, although the results were not significant in any of these stages (*p* > 0.05). No significant differences were established between women’s different age strata and maternal satisfaction. However, the average given by women regarding their satisfaction with the process, on a scale from 0 to 4, was: 3.5 ± 0.5 in general, 3.2 ± 0.8 regarding pregnancy, 3.7 ± 0.5 in childbirth and 3.1 ± 0.6 in the postpartum period. The woman’s satisfaction with the follow up and health care received during pregnancy, childbirth and puerperium is independent of the mother’s age.

## 1. Introduction

Satisfaction with health care services is a complex concept that is related to a large variety of factors, including lifestyle, previous experiences, future expectations, and individual and societal values [1]. This multidimensional concept integrates different aspects such as communication, professional competence, accessibility and availability, time dedicated to the user, efficiency, continuity, and physical environment [2], and is influenced by socio-demographic characteristics, the user’s health status, and satisfaction with professional development [3]. When improving health care, user opinions and expectations are increasingly taken into account. One of the factors most taken into account in recent years is the satisfaction of users with the care received. This variable is especially taken into consideration in the process of care in special situations such as pregnancy and childbirth [4]. The model of perinatal care promulgated by the World Health Organization (WHO) encourages the participation of women, so that they are the protagonists of their delivery and that they feel satisfied with the care received during their delivery. [5]

Today’s fertility rates are reflected by cultural changes, changes related to the labor market, expectations toward the health system related to aspects of health promotion and quality of care, as well as factors related to the limited intervention of public authorities in terms of family maintenance, which, among others, relegates motherhood to the background [6,7].

A woman’s desire to have a child after age 35 and even 40 years has become an important social phenomenon and, in daily clinical practice, an increase in pregnancy in women over 35 years of age has been observed [8].

In Spain, according to INE (National Institute of Statistics, Spain) data, there were 369,302 births in 2018—of which, 147,942 were in women 35 years of age or older, representing 40.06% of the total births. The age at which the highest number of births occurred was 34 [9].

The association of maternal age with care in pregnancy and childbirth has been little studied [10,11,12,13]. In a study carried out in Italy with 277 women, no association was found between maternal satisfaction with care received during delivery and socio-demographic variables, including the age of the mother [12]. In a study in Kenya, women were found to be satisfied with the care received in prenatal care; however, a difference associated with the age of the mother was not evaluated [13].

The number of women who become mothers at an older age is high, and few studies have been identified that associate maternal satisfaction with pregnancy and childbirth care with the mother’s age. These existing studies identify the need for further studies investigating this relationship. We think that the age at which the mother becomes a mother for the first time influences the important parameter in perinatal care, maternal satisfaction—a parameter increasingly taken into account by health care systems. Therefore, we proposed the objective of evaluating the effect of maternal age on maternal satisfaction of the health care received during each stage of pregnancy, childbirth and puerperium process, and also their global satisfaction with the entire process.

## 2. Materials and Methods

An observational, analytical, multicenter study was conducted in the following five hospitals in the Andalusian public health system: Jaen Hospital Complex in Jaén, Hospital San Agustín in Linares (Jaén), Hospital San Juan de la Cruz in Úbeda (Jaén), Hospital Virgen de las Nieves in Granada, and Hospital Reina Sofía in Cordoba. The study was conducted from May 2016 to May 2018.

The reference population was primiparous pregnant women with a singleton gestation and 18 years old or older.

Mothers who had difficulty communicating in Spanish were excluded (language barrier).

### 2.1. Sample Size

The sample size was calculated for the principal objective of the study: the appearance of any pathology during pregnancy. For this calculation, the study by Heras Pérez et al. 2011 [14] was used as reference. According to this study, the incidence of pregnancy-associated pathology in woman over the age of 35 years was 29.2% compared with 15.8% in women 35 years old or younger; and to achieve a power of 80% to detect differences in the null hypothesis H₀:μ₁ = μ₂ using a bilateral chi-squared test for two samples with a significance level of 5%, a sample size of 302 women was needed. After taking into account a drop-out rate of 15%, 373 women were recruited. Women were selected consecutively, with a sample size proportional to the specific weight of each center.

### 2.2. Data Collection

Data were collected with a previously piloted questionnaire that was heteroadministrated by qualified personnel with expertise on this topic (in which the midwife completes the questionnaire after asking the woman the questions). The questionnaire was administered via an interview two hours following childbirth and at hospital discharge, and at two months postpartum via a telephone call. The majority of data were obtained via the clinical interview and during the telephone call made by the health professional following childbirth; however, data were completed with access to the clinical history and the pregnancy health document.

Information was collected on the following variables: socio-demographic, obstetric antecedents, lifestyle, pregnancy, childbirth, the puerperium, the newborn, psychological aspects, health care provider, and the level of satisfaction with the care received throughout the whole process.

### 2.3. Data Analysis

Continuous variables were evaluated by comparison of means, t-test, or analysis of variance. The analysis of covariance was used to estimate means adjusted for possible confounding factors. Confounding factors were considered as those variables that were not intermediate variables and modified the co-efficient of the principal exposition (maternal age) by more than 10% in the multivariate models.

### 2.4. Ethical Considerations

This study was approved by the Research Ethics Committee of the hospitals that participated in this study (Acta n° 247, ref. 3016). Informed consent was obtained from all participating women, and the established protocols for the respective health centers were followed for access to medical record data and to be able to conduct this type of study with the intention of publication/dissemination to the scientific community.

## 3. Results

A total of 373 women participated. The mean age of the participating women was 30.45 ± 5.63 years. In terms of civil status, 62.2% (232) were married. Almost all, 98.1% (366), were Spanish. Eight (2.1%) had no formal education level, 6.7% (25) had primary level education, 53.4% (199) had secondary level education, and 37.8% (141) had university level education. The mean income level of 36.7% (137) was between 1000 and 1999 euros monthly, followed by 26.3% (98) with a mean income lower than 1000 euros, and 8.3% (31) with a mean income 3000 euros or higher. During pregnancy, 71% (265) were employed, and 12.4% (26) had some type of pathology prior to pregnancy. A previous history of miscarriage was reported in 24.9% (93). Pregnancy was planned in 87.4% (326) and required medical assistance to achieve pregnancy in 12.3% (46). Pregnancy care was received in the public health care system in 48.8% (182), 49.3% (184) alternated between the public and private health care system, and 1.9% (7) chose only to receive care in the private health care system. Over half (58.4%) of the women attended antenatal education. The mean gestational age at birth was 39.43 ± 1.41, as shown in Table 1, in which the characteristics of the study population are described.

Table 2 presents maternal satisfaction with the care received during pregnancy, childbirth, and the puerperium according to maternal age. No significant association was found between maternal age and her satisfaction with the process of care during pregnancy, childbirth, and the puerperium. Nevertheless, it can be noted that the evaluations of satisfaction with the care received in general were mostly quite satisfied or very satisfied. In terms of satisfaction during pregnancy, 43.01% of the sample were very satisfied, whereas 74.25% felt very satisfied with the care received during childbirth. During the puerperium, the majority, 60.48%, felt quite satisfied. None of the women indicated that they were not at all satisfied with the care received during childbirth, nor during the puerperium, nor for global satisfaction for the process of pregnancy, childbirth, and the puerperium. Comparing the different levels of satisfaction that were established with the lowest level that had been considered in each of the variables, no significant differences were found (*p* > 0.05).

Table 3 shows the association between maternal age and satisfaction with the process of care during pregnancy, childbirth, and the puerperium stratified by age. No significant differences were observed between the different age groups of women and maternal satisfaction. Nevertheless, the mean evaluation by the women regarding their satisfaction with the stages, on a scale of 0 to 4, was: 3.56 ± 0.52 in general (*p* = 0.935), 3.23 ± 0.80 for pregnancy (*p* = 0.483), 3.71 ± 0.51 for childbirth (*p* = 0.751) and 3.12 ± 0.66 for the puerperium (*p* = 0.360).

## 4. Discussion

Satisfaction with the care received during the stages of pregnancy, childbirth, and the puerperium was not shown to be related to maternal age, according to the results of the present study.

Along the same lines, Sánchez Fortis et al., after conducting a bibliographic review with 27 articles to identify the overall satisfaction of women during childbirth care and analyzing the factors that influenced this, concluded that the most relevant aspects in the satisfaction of women with childbirth are: being accompanied during childbirth by their partner or a family member, the support received from health care professionals, receiving detailed and understandable information on the process which assists decision making, as well as the expectations of the women regarding childbirth and, most importantly, in terms of pain relief. Among their results, the influence of maternal age on satisfaction with childbirth care did not stand out [10].

Similarly, Martínez-Galiano conducted a prospective multicenter cross-sectional study including 507 women who gave birth in different hospitals in the provinces of Granada, Almería, and Jaen (Spain) during 2011 and 2012, which aimed to evaluate the influence that attending antenatal education sessions can have on certain aspects related to the process of pregnancy, childbirth and the puerperium, among which was the woman’s satisfaction with the care received during pregnancy and the care received during childbirth. Martínez-Galiano concluded that antenatal education had no influence on the satisfaction that women report for the process of care during pregnancy, childbirth and puerperium (*p* > 0.05); moreover, when adjusting for maternal age, among other variables, no changes were observed in this association [11].

In contrast to our results, there have been authors who have established significant relationships between the mother’s age and maternal satisfaction with the pregnancy, childbirth, and puerperium care process and also with the globally assessed care process [2,3,15,16].

Pozo-Cano, in his cross-sectional descriptive work evaluating the satisfaction of women with the care received during pregnancy and childbirth in 429 postpartum women who gave birth in two hospitals of the Andalusian Public Health System in Granada city from April to July 2012, did not find significant differences between satisfaction with pregnancy control and socio-demographic variables. However, of the ten dimensions that integrate satisfaction with childbirth care, she found significant differences in the dimension that analyzes the environment, and observed that the lower the age of women, the higher the level of satisfaction they had and vice versa (*p* = 0.018) [3]. These results do not coincide with those obtained in other studies that state that younger women report a lower level of satisfaction than older women, because, in general, although younger women may participate more in the process of pregnancy, they are also less conformist [2].

Tierra-Burguillo, in his mixed study on women registered with Huelva capital health centers who had children aged between 12 and 24 months exploring the level of satisfaction for maternal and child health education, described the relationship between the degree of satisfaction and maternal socio-demographic characteristics and other variables related to parenting. This study observed the following variables with statistical significance: being employed, attending maternal health education workshops, being physically active, choice of breastfeeding, and maternal age. He stressed that the satisfaction of mothers’ maternal and child health education increased linearly with maternal age; the older the mother, the higher the level of satisfaction (*p* = 0.013) [15].

The high satisfaction of these women may be due to the good maternal and neonatal outcomes: there was no maternal or neonatal mortality [17,18]. 

### Limitations

The sample is representative of the population. The questionnaire used for data collection was piloted previously. The questions were formulated to be clear and understandable for all education levels, making an information bias unlikely. We can never entirely rule out a memory bias but, a priori, its influence on the results will have been insignificant because of the type of information that is collected and the short period of time during which the information was collected. A selection bias associated with non-response is unlikely to have had an influence on the results, as the majority of women were recruited, with only 13 refusing to participate. There was nothing to suggest that those who did not respond were different from those that did. A possible confounding bias has been addressed, although it is not possible to completely rule out the confounding bias inherent to observational studies. Confounding factors as well as some variables were selected according to the literature review, which, due to the experience of the researchers, a priori, could influence the results. For example, a woman with pathology prior to pregnancy may receive more comprehensive pregnancy care, and this may influence her level of satisfaction with the care received

Overall, women reported satisfaction (quite satisfied) with the follow up and health care received during the process of pregnancy, childbirth, and the puerperium. This evaluation is independent of maternal age.

## Figures and Tables

**Table 1:** Characteristics of the study population (n = 373).

Variable	Value
Age, mean (SD)	30.45 (5.63)
Civil status, n (%)	
Single	102 (27.3)
Married	232 (62.2)
De facto relationship	36 (9.7)
Divorced	3 (0.8)
Nationality, n (%)	
Spanish	366 (98.1)
Other	7 (1.9)
Education level, n (%)	
None	8 (2.1)
Primary	25 (6.7)
Secondary	105 (28.2)
Baccalaureate/Professional development *	94 (25.2)
Tertiary	141 (37.8)
Income level, n (%)	
<1000 Euros	98 (26.3)
1000–1999 Euros	137 (36.7)
2000–2999 Euros	68 (18.2)
≥3000 Euros	31 (8.3)
Employed during pregnancy, n (%)	
No	108 (29.0)
Yes	265 (71.0)
Presence of illness, n (%)	
No	326 (87.6)
Yes	46 (12.4)
Previous miscarriage, n (%)	
No	280 (75.1)
Yes	93 (24.9)
Planned pregnancy, n (%)	
No	47 (12.6)
Yes	326 (87.4)
Antenatal education, n (%)	
No	155 (41.6)
Yes	218 (58.4)
Pregnancy care, n (%)	
Public health care system	182 (48.8)
Private health care system	7 (1.9)
In both	184 (49.3)
Medical assistance to achieve pregnancy, n (%)	
No	327 (87.7)
Yes	46 (12.3)
Gestational age at birth, mean (SD)	39.43 (1.41)

* Equivalent of A levels/upper secondary level education.

**Table 2:** Influence of maternal age on the woman’s satisfaction with the process of pregnancy, childbirth, and the puerperium.

Variable	Total, n	Crude Analysis		Multivariate Analysis	
		Age *m, sem*	*p*-Value	Age m*, sem	*p*-Value
Satisfaction—pregnancy			0.615		
Not at all	1	36.40 ± 0		36.88 ± 5.06	ref.
A little satisfied	9	31.62 ± 1.35		32.55 ± 1.70	0.417
Satisfied	53	30.04 ± 0.69		29.66 ± 0.74	0.158
Quite satisfied	149	30.80 ± 0.50		30.52 ± 0.45	0.211
Very satisfied	160	30.20 ± 0.44		30.12 ± 0.42	0.184
Satisfaction—childbirth			0.548		
Not at all	0				
A little satisfied	0				
Satisfied	11	28.66 ± 1.66		29.03 ± 1.53	ref.
Quite satisfied	84	30.65 ± 0.68		30.44 ± 0.60	0.392
Very satisfied	274	30.44 ± 0.33		30.28 ± 0.32	0.427
Satisfaction—puerperium			0.341		
Not at all	0				
A little satisfied	7	33.34 ± 1.74		31.93 ± 2.06	ref.
Satisfied	40	31.28 ± 0.96		31.15 ± 0.86	0.73
Quite satisfied	225	30.36 ± 0.37		30.19 ± 0.35	0.407
Very satisfied	100	30.03 ± 0.55		29.92 ± 0.54	0.348
Satisfaction—general			0.253		
Not at all	0				
A little satisfied	1				
Satisfied	5	31.69 ± 0.92		29.67 ± 2.57	ref.
Quite satisfied	152	30.96 ± 0.47		30.67 ± 0.43	0.703
Very satisfied	215	30.02 ± 0.38		29.97 ± 0.36	0.909

* Adjusted for maternal education level, income level, maternal smoking habit, previous history of miscarriage, and the presence of any pathology prior to pregnancy.

**Table 3:** Association between maternal age and satisfaction with the process of pregnancy, childbirth, and the puerperium stratified by age.

Variable	Total, n *m ± SD*	Crude Analysis					Multivariate Analysis				
		Age (Years) *m ± sem*				*p* Value	Age (Years) *m* * *± sem*				*p* Value
		<25	25–29	30–34	≥35		<25	25–29	30–34	≥35	
Satisfaction—pregnancy (0–4)	3.23 ± 0.80	3.33 ± 0.10	3.22 ± 0.09	3.21 ± 0.07	3.22 ± 0.08	0.809	3.34 ± 0.13	3.23 ± 0.09	3.22 ± 0.07	3.21 ± 0.09	0.483
Satisfaction—childbirth (0–4)	3.71 ± 0.51	3.67 ± 0.08	3.66 ± 0.06	3.80 ± 0.04	3.67 ± 0.05	0.131	3.68 ± 0.08	3.65 ± 0.06	3.80 ± 0.05	3.67 ± 0.06	0.751
Satisfaction—puerperium (0–4)	3.12 ± 0.66	3.24 ± 0.09	3.18 ± 0.07	3.10 ± 0.06	3.05 ± 0.07	0.314	3.27 ± 0.10	3.16 ± 0.07	3.07 ± 0.06	3.07 ± 0.07	0.360
General satisfaction (0–4)	3.56 ± 0.52	3.65 ± 0.07	3.61 ± 0.06	3.53 ± 0.05	3.53 ± 0.05	0.364	3.63 ± 0.08	3.63 ± 0.06	3.52 ± 0.05	3.54 ± 0.06	0.935

* Adjusted for maternal education level, income level, maternal smoking habit, previous history of miscarriage, and the presence of any pathology prior to pregnancy.
